# Unencapsulated and washable two-dimensional material electronic-textile for NO_2_ sensing in ambient air

**DOI:** 10.1038/s41598-022-16617-1

**Published:** 2022-07-19

**Authors:** Pelumi W. Oluwasanya, Tian Carey, Yarjan Abdul Samad, Luigi G. Occhipinti

**Affiliations:** 1 Cambridge Graphene Centre, Department of Engineering, University of Cambridge, Cambridge, UK; 2 CRANN and AMBER Research Centres, Trinity College Dublin, Dublin, Ireland

**Keywords:** Electrical and electronic engineering, Mechanical engineering

## Abstract

Materials adopted in electronic gas sensors, such as chemiresistive-based NO_2_ sensors, for integration in clothing fail to survive standard wash cycles due to the combined effect of aggressive chemicals in washing liquids and mechanical abrasion. Device failure can be mitigated by using encapsulation materials, which, however, reduces the sensor performance in terms of sensitivity, selectivity, and therefore utility. A highly sensitive NO_2_ electronic textile (e-textile) sensor was fabricated on Nylon fabric, which is resistant to standard washing cycles, by coating Graphene Oxide (GO), and GO/Molybdenum disulfide (GO/MoS_2_) and carrying out in situ reduction of the GO to Reduced Graphene Oxide (RGO). The GO/MoS_2_ e-textile was selective to NO_2_ and showed sensitivity to 20 ppb NO_2_ in dry air (0.05%/ppb) and 100 ppb NO_2_ in humid air (60% RH) with a limit of detection (LOD) of ~ 7.3 ppb. The selectivity and low LOD is achieved with the sensor operating at ambient temperatures (~ 20 °C). The sensor maintained its functionality after undergoing 100 cycles of standardised washing with no encapsulation. The relationship between temperature, humidity and sensor response was investigated. The e-textile sensor was embedded with a microcontroller system, enabling wireless transmission of the measurement data to a mobile phone. These results show the potential for integrating air quality sensors on washable clothing for high spatial resolution (< 25 cm^2^)—on-body personal exposure monitoring.

## Introduction

Millions of premature deaths worldwide have been linked to bad air quality indoors and outdoors^1,2^. Epidemiological studies maintain that exposure to pollutant levels beyond the prescribed limits (40 µg m^−3^—annual mean value for NO_2_) could have lethal consequences, especially on children^3,4^, pregnant women^5,6^ and the elderly^7^. Undesired effects to the cardiovascular system are evidenced by the observed relationship between hospital admission data and emergency room visits and air pollution data for the same/overlapping timeline^8,9^. Air pollution data are mainly collected from a minimal number of air quality monitoring sites^10^ or ad hoc networks^11^ (i.e. a network that is composed of devices communicating with each other), installed at fixed locations with spatial resolution of several hundred meters in the best case, mostly of a few kilometers in urban areas and hundreds of kilometers in rural ones. Due to the spatial sparseness, the data collected can be different from actual individual exposure levels throughout a day^12^. Wearable sensors can solve this problem as they exist in the local environment of the subject. However, for reasons of convenience and utility, the problem must be solved non-intrusively. Next-generation wearable sensors and electronics aim to become embedded into the user's clothes to achieve ultimate comfortability for the user to wear. Technologies currently exist to address portable gas sensing include on-body patches based on stretchable polymers^13^ or rigid silicon-based devices encased in a box that can be stuck to textile^10^. On-body patches are limited by low breathability and skin compatibility issues associated with adhesives or elastic bands adopted in the patches to ensure wearability, often causing skin irritation and discomfort to the user. At the same time, conventional silicon-based electronics is generally bulky and intrusive as it was not originally designed to conform to a textile surface. When used as substrates for electronic sensors, textile materials possess all the desirable attributes for wearable sensor applications, such as high flexibility, bio/skin compatibility, breathability, conformability to arbitrary shape and size, proximity to the measurement site, and can be worn by the user for long periods without causing discomfort. Sensors that have been successfully integrated on textiles include temperature sensors, potentiometric sensors^14^, tactile sensors^15^, humidity sensors^16,17^, capacitive^18^, strain gauge and pressure sensors^19,20^. Direct fabrication and coating methods of active sensing materials on fabrics, based on dip-coating^18^, chemical reduction^19,21^, hot pressing^19^ and printing^22^ have had issues such as uniformity of coating, skin compatibility and poor washability, due to inability of the coated material to form strong bonds with the fibres of the fabric^23^. Attempts to overcome this limitation have been reported by fabricating textile-based sensors directly on fibres and yarns employing a controlled coating of the sensing material on the fibres of the yarn^24^. The yarns are then woven together and integrated into smart clothing^25,26^ or non-clothing^27^ textile systems. Sensitivity and selectivity of the fabricated sensor depends on the active material properties. While two-dimensional (2D) materials such as graphene and related materials with high surface area (theoretically 2630 m^2^ g^−1^) show very high sensitivity to low concentrations of NO_2_ (down to ppb level), as demonstrated by Yuan et al*.*—150 ppb^28^, Liu et al*.*—5 ppb^29^, Fowler et al*.*—5 ppm^30^, Shaik et al*.*—2.5 ppm^31^, Wang et al*.*—5 ppm^32^, Novikov et al.—1 ppb^33^ their investigation for gas sensing has been limited to single yarns that may then be woven into fabric^26^, or encapsulated. Graphene-based sensors have been demonstrated with sensitivity up to 250 ppb^26^. Several works have also been undertaken with transition metal dichalcogenides on Si/SiO_2_ for NO_2_ detection with low LOD such as MoS_2_, Tungsten disulfide (WS_2_) and Tin disulfide (SnS_2_) due to their ability to operate in low temperature (100–150 °C)^34^. For example chemical vapor deposition MoS_2_ with graphene has been used with to enable an optoelectronic gas sensor achieving a LOD of 0.1 ppb and sensitivity of 4.9%/ppb^35^. Yang et al.^36^ demonstrated liquid phase exfoliated (LPE) SnS_2_ gas sensor with a sensitivity of 0.3%/ppm with a LOD of 50 ppm while Ko et al.^37^ used atomic layer deposition WS_2_ with silver nanowires on Si/SiO_2_ to achieve a sensitivity of 0.1%/ppm. In spite of the extensive work published so far on graphene and 2D materials as reviewed e.g., by Buckley et al.^34^. NO_2_ gas sensing on textile substrates has not been achieved with high sensitivity, low LOD and low operating temperature (< 150 °C). Moreover, a protocol to enable washable NO_2_ gas sensors with transition metal dichalcogenides is yet to be demonstrated without the use of encapsulation layers.

In this work we demonstrate that our proposed textile gas sensors are able to maintain or even improve their sensing performance over International Standard Organization (ISO) standard washing cycles in absence of any encapsulation layers.

We report a flexible, lightweight and biocompatible gas sensor, which is integrated entirely into the textile material. The sensor is selective to the concentration of NO_2_ in a gas mixture, both in dry and humid air. The sensor resistance changes by 28% when exposed to 2 ppm of NO_2_, compared to 6.5% for 40,000 ppm of CO_2_ and 0% for 2 ppm of NH_3_. Low concentrations of NO_2_ (as low as 20 ppb) change the sensor’s resistance by 1.4% in dry air, corresponding to a LOD of 7.3 ppb. This sensitivity value is an order of magnitude greater than the state-of-the-art e-textile gas sensors with 2D materials in dry air^26^. A 100 ppb concentration of NO_2_ in > 60% relative humidity at room temperature changes the sensor resistance by 3.04%.

We show that the sensor is selective to NO_2_ in the presence of NH_3_, CO_2_ and humidity, and demonstrates the resilience of the e-textile sensor for at least 100 cycles of ISO-standard washes. Selectivity is achieved at ambient temperature (20 °C). This temperature is significantly lower than the high operating temperatures typically needed for metal oxide gas sensors (e.g. SnO_2_ at 400 °C)^34^. Finally, we couple the e-textile gas sensor with a micro-controller for a real-time data collection and monitoring system. Using a supply voltage of only 3 V, the sensor response (change in resistance) can be measured and wireless transmitted e.g. via Bluetooth^®^. Integrating the proposed sensor devices with a mobile platform for data collection and real-time monitoring is achieved and is an essential step enabling personalized, high resolution (< 25 cm^2^), remote monitoring of air quality levels.

## Results

**Figure 1:**
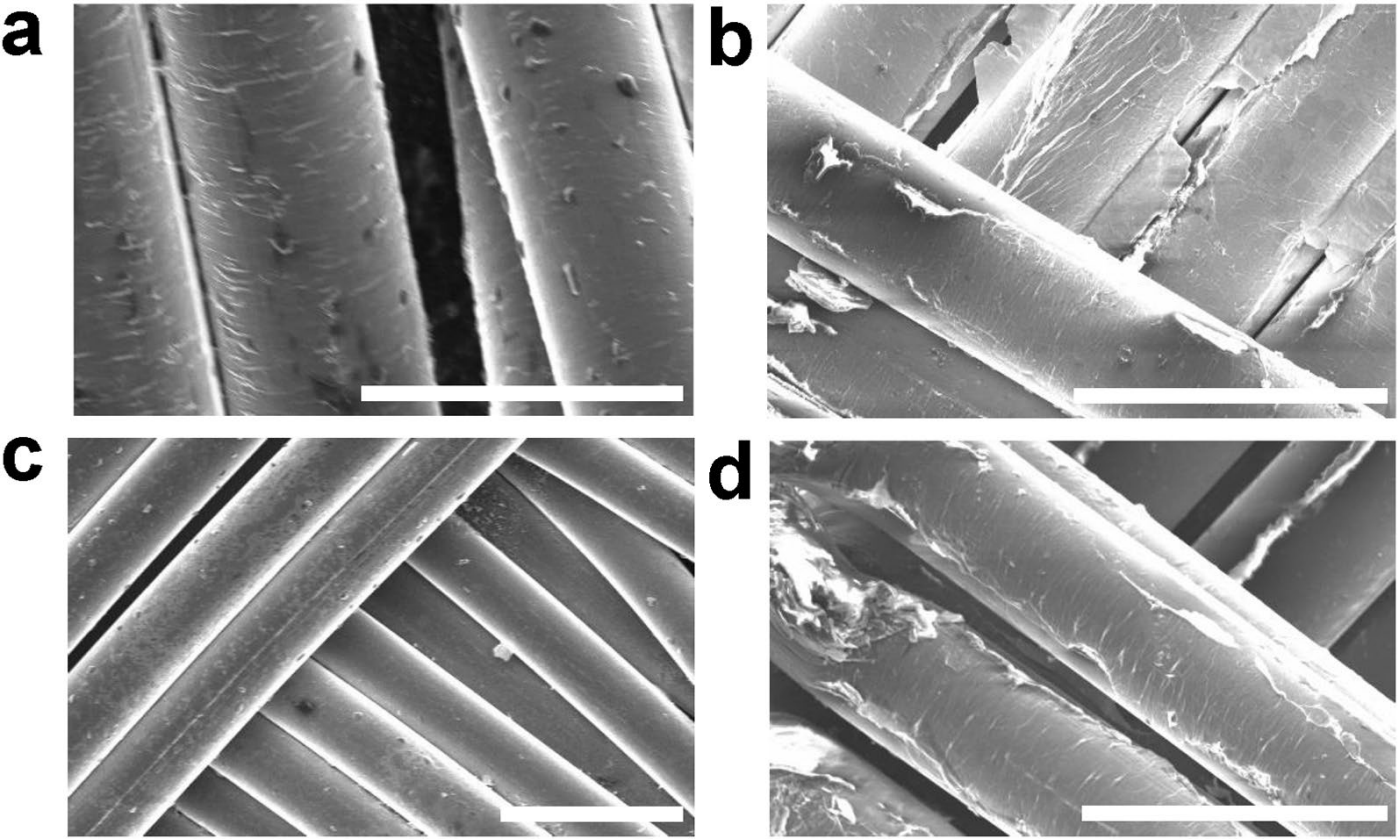
Investigation of fabric morphology. SEM images showing the coated fabric without washing (**a**, **c**), and after 100 cycles of ISO-standard wash (**b**, **d**) for the RGO and RGO/MoS_2_ coated fabric, respectively. The scalebars are (**a**) 30 µm, (**b**) 40 µm, (**c**) 50 µm and (**d**) 40 µm respectively.

**Figure 2:**
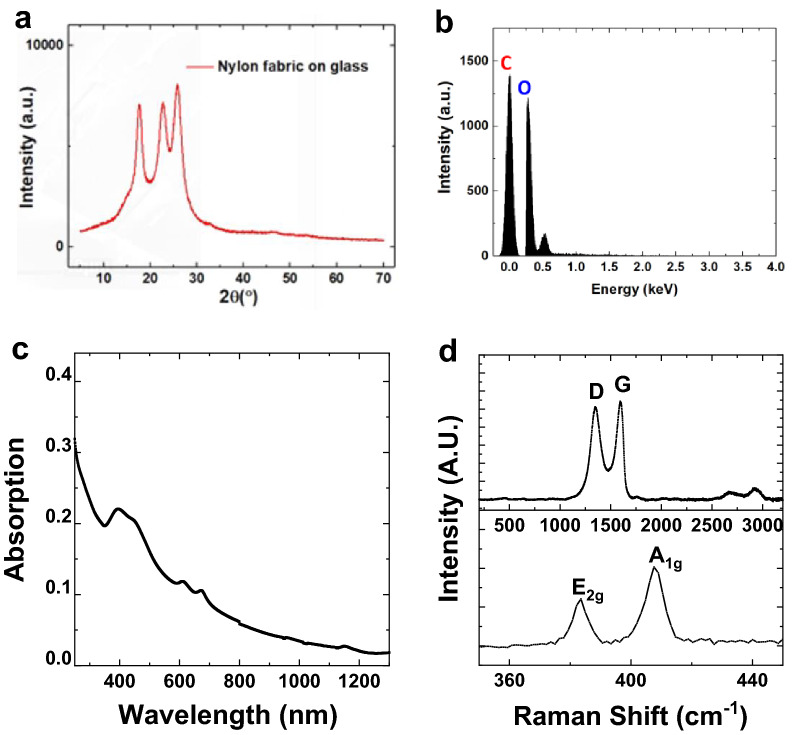
Characterisation of textile and electronic inks. (**a**) XRD of nylon fabric on glass substrate. (**b**) Energy Dispersive X-ray analysis of RGO-coated fabric. (**c**) UV–Vis of the MoS_2_ ink with four absorption peaks. (**d**) Raman Spectroscopy of the MoS_2_ ink and RGO.

The GO and GO/MoS_2_ e-textile sensors were fabricated by a modified dip-coating method. We use MoS_2_ to give the sensor selectivity, while we use GO to increase the sensor's conductivity to read an electrical signal easily. The GO was partially reduced thermally at 170 °C in an oven after deposition on Nylon. This process created the RGO (Fig. 1a) and RGO/MoS_2_ (Fig. 1c) e-textiles. In Fig. 1a,c, we use scanning electron microscopy (SEM) to show a uniform fabric coating with RGO and RGO/MoS_2,_ respectively. The RGO and RGO/MoS_2_ coating on the Nylon fabric and the fabric itself appear to remain unaffected by the heat as shown in the SEM images (Fig. 1a,c). The applied heat likely created strong adhesion between the layer of RGO and the fibres of the fabric^38^. We attribute the improved adhesion to hydrogen bonding between the RGO functional group on the edges of the flakes and the fibre^19^ surface and the thermally induced adhesion of thin RGO layers on Nylon surface^39^. Single yarns, for example, in Fig. 1a,d show tiny streaks of folds (wrinkles) in the coating layer likely due to the thermal stress on the layer during the reduction of the RGO as well as the rough surface of the yarns. Figure 1b,d show an increase in the folding and subsequent tearing of the ‘mat’-like folds due to the mechanical stress experienced after washability testing. We characterize the fabric using X-ray diffraction (XRD), Fig. 2a is the XRD of the blank Nylon fabric on glass shows sharp diffraction peak at 2θ = 17.6°, and a merged one (due to amorphous glass) at 22.7° and 25.8° depicting crystalline packing due to inter-polymer hydrogen bond^40^. Elemental compositional analysis carried out on the GO coated and annealed fabric using Energy Dispersive XRay (EDX) analysis (Fig. 2b) showed an O_2_ contribution of 22.82%wt, which is in agreement with Morimoto et al*.*^41^, for GO reduced at 170 °C. Ultraviolet–visible spectroscopy (Fig. 2c) was used to estimate the flake concentration *c* in the MoS_2_ ink using the Beer–Lambert law which correlates the absorbance *A* = *αcl*, to the absorption coefficient *α*, the flake concentration *c*, and the light path length *l*. The MoS_2_ ink is diluted 1:200 with water/SDC and placed in a cuvette 1 cm in length. We find concentration of MoS_2_ (*C*_MoS2_) ~ 0.56 mg ml^−1^ when an absorption coefficient of *α*_MoS2_ ~ 3400 L g^−1^ m^−1^ at 660 nm is used^42^. The concentration is consistent with previous 2D material inks prepared via sonication^43^. The spectra of the MoS_2_ ink displays four characteristic peaks at 309 nm, 445 nm, 609 nm and 673 nm that are attributed to the excitonic transitions of MoS_2_ transition metal dichalcogenide flakes^44^. The GO ink was a commercial water-based ink and had a GO concentration of 4 mg ml^−1^. The concentration of the GO/MoS_2_ ink was 3.68 mg ml^−1^ after mixing. This corresponds to a weight ratio of GO/MoS_2_ of about 70:1.

In Fig. 2d, Raman spectroscopy (Renishaw 1000 InVia) is used to examine the defects and thickness of the GO and MoS_2_ flakes. The MoS_2_ flakes show the A_1g_ (407 cm^−1^) and E_2g_ (383 cm^−1^) peaks typical of multilayer MoS_2_^45^. The small frequency (24 cm^−1^) difference between A_1g_ and E_2g_ confirms the multilayer (> 4 layer) nature of the flakes^46^. The Raman spectra of the RGO shows a D peak at about ~ 1350 cm^−1^ and a G peak located at about ~ 1600 cm^−1^. The I(D)/I(G) ratio is ~ 0.95 which is typical of a highly defective basal plane due to the presence of functional groups^19^.

**Figure 3:**
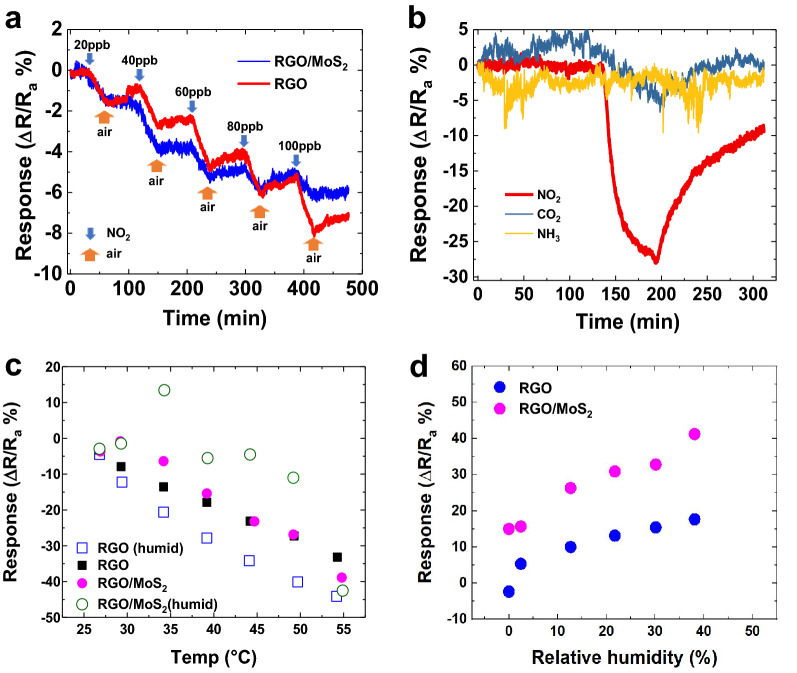
Textile gas sensing. (**a**) Response of fabricated sensors to increasing NO_2_ concentrations in dry air from 20 to 100 ppb for RGO-coated fabric and RGO/MoS_2_-coated fabric respectively. Both sensors show little recovery in dry air. (**b**) Gas sensing response of the RGO/MoS_2_-coated fabric to NO_2_, NH_3_ and CO_2_ showing selectivity of the coated material to NO_2_. (**c**) Variation in response of the RGO and RGO/MoS_2_-coated fabric due to increasing temperature at a fixed concentration of 100 ppb NO_2_ both in dry air and humid air respectively. (**d**) Variation in response of the RGO and RGO/MoS_2_-coated fabric due to increasing humidity at a fixed concentration of 100 ppb NO_2_ helps determine the humidity correction relationship.

The RGO and RGO/MoS_2_ sensors were exposed to increasing concentrations of NO_2_ from 20 to 100 ppb in dry air (Fig. 3a). For a 20 ppb concentration of NO_2_ RGO-coated & RGO/MoS_2_-coated fabrics showed a ~ 1.5% change in electrical resistance relative to their original resistances corresponding to a sensitivity of ~ 0.08%/ppb. We define the limit of detection (LOD) as the smallest concentration of gas that can be detected LOD ≥ 3σ/S, where σ is the noise level (i.e., standard deviation of the response) in the absence of the analyte gas and S is the sensitivity. We find an LOD of 2.7 ppb for the RGO-coated fabric and 7.3 ppb for the RGO/MoS_2_-coated fabric. The LOD is one of the lowest recorded values 2D material gas sensing^34^.

This response was calculated from:^47^

$$\frac{\Delta R}{{R}_{a}}(\%)= \frac{{R}_{g}- {R}_{a}}{{R}_{a}} \times 100\%$$

where ${R}_{g}$ is the resistance of the material in the analyte gas, ${R}_{a}$ is the resistance of the material in air $\Delta R$ and is the difference ${R}_{g}- {R}_{a}$.

Gas sensing with RGO and RGO/MoS_2_ is due to the adsorption of gas molecules on the material's surface^48^. Upon adsorption of the gas molecule, electrons are transferred between the gas molecules and the 2D material^49^. For example, NO_2_ will donate electrons to MoS_2_ and accept electrons from RGO, altering the electronic properties of the sensor, which can be seen as a resistance change^50,51^. Thus the RGO and RGO/MoS_2_ can detect the presence of NO_2_ molecules. To test for selectivity, we exposed the unwashed sensor to 2 ppm NO_2_, 2 ppm NH_3_ and 40,000 ppm CO_2_ in humid air. The response to 2 ppm NO_2_ was 28%, and 6.5% to for the 40,000 ppm CO_2_ and no measurable response to 2 ppm NH_3_ (Fig. 3b). Hence the RGO/MoS_2_ sensor demonstrates orders of magnitude (~ 3000 times) more selectivity to NO_2_ than CO_2_ and is completely selective to NO_2_ when compared to NH_3_. Theoretical work based on density functional theory agrees with our findings as it is predicted that induced charge transfer between NO_2_ and MoS_2_ (~ 0.06 e) is more significant than for CO_2_ and NH_3_ (~ 0.02 e)^50,51^.

The effects of temperature on the sensor sensitivity were investigated in both dry and humid air conditions (Fig. 3c). In dry air, RGO-coated fabric sensor response increased from − 3.30 to − 33.19% for a temperature rise from 26.80 to 54.29 °C (i.e. ~ 1.32%/°C), while for humid air, it also increased from − 4.5 to − 44.0% for the same temperature range (i.e. ~ 1.44%/°C). The RGO/MoS_2_-coated fabric response also increased from − 3.60 to − 38.90% in dry air (i.e. ~ 1.28%/°C). In humid air, it increased from − 2.90 to − 42.53% (i.e. ~ 1.44%/°C).

**Figure 4:**
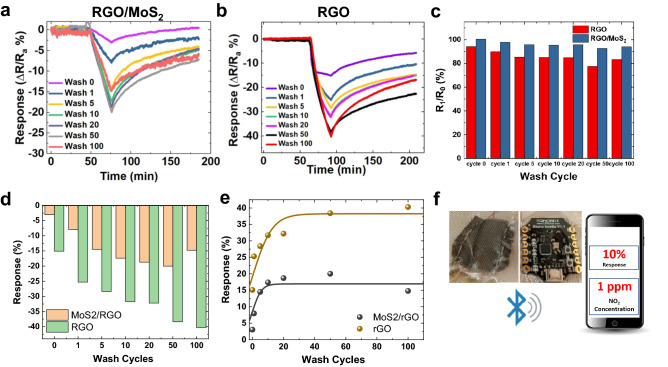
Textile gas sensing in humid air with washing. (**a**) Gas sensing response of the RGO/MoS_2_-coated and (**b**) RGO-coated fabric to 100 ppb NO_2_ in humid air. The change in resistance expressed as a percentage for the fabric before exposure to NO_2_, during exposure to NO_2_, and after exposure to NO_2_—with partial recovery. (**c**) Recovery of the RGO and RGO/MoS_2_-coated fabric after exposure to a fixed concentration of 100 ppb NO_2_ after 2 h. (**d**, **e**) show the sensor response dependency of the RGO/MoS_2_ and RGO coated fabric on the number of washing cycles. (**f**) Device integration showing the sensor integrated to fabric, the micro-controller and the mobile application.

The RGO-coated fabric showed an increasing $\Delta R/R$ with relative humidity (Fig. 3d). For example, the sensor response increased from − 2.3 to 17.65% when RH increased from 0.0 to 38.1% (i.e. ~ 0.45%/%RH). Upon testing the RGO/MoS_2_-coated fabric with increasing humidity from 0.0 to 38.1%RH, it showed a higher response than the RGO-coated fabric with steady response from 15 to 41.23% (i.e. ~ 0.69%/%RH). This is consistent with the expected response of a chemiresistive sensor is better in moisture than in dry air due to the positive contribution of moisture to the gas absorption in the sensing mechanism^52^. In a real-world environment where there is a likelihood of a mixed environment scenario (i.e. NO_2_ mixed with humidity) our sensor could potentially be coupled with machine learning based on statistical data processing as recently demonstrated for metal oxide sensors operating in gas mixtures^53^. Gas sensing results in humid air as a function of washing are shown in Fig. 4a,b,d,e. With the sensor at steady state before exposure, response of the un-washed RGO-coated fabric to 100 ppb NO_2_ was 15.1% (Fig. 4b,d,e) while that of the unwashed RGO/MoS_2_-coated fabric was 3.04% (Fig. 4a,d,e). The exposure period was 30 min in all cases. There was a considerable increase in response in both coating types after a single cycle wash. RGO-coated fabric showed 67.5% increase in sensitivity as the recorded change in resistance was 25.3% while the RGO/MoS_2_-coated fabric showed a 161% increase in sensitivity as sensor response jumped to 7.96%. This increase after the first cycle was the most significant increase for both coating types. The RGO-MoS_2_-coated fabric showed 82.0% increase after 5 wash cycles with a sensor response of 14.5% while the RGO-coated fabric sensitivity to NO_2_ increased by 12.2% with a sensor response of 28.4%. After 10 and 20 cycles, the RGO-coated fabric showed very little increase in response, increasing from 28.4% for 5 cycles to 31.7% with a sensor response of 32.2% respectively for 10 and 20 cycles. Whereas the sensor response of RGO/MoS_2_-coated fabric was 17.4% and 18.7% for 10 and 20 wash cycles, respectively. After 50 wash cycles, the response of the RGO-coated fabric had increased to 38.4%, while that of the RGO/MoS_2_-coated fabric was 20%. However, the response of the RGO/MoS_2_-coated fabric dropped by 26% with a sensor response of 14.8% when subjected to a further 50 wash cycles while the response of the RGO-coated fabric increased by a further 5% as the recorded sensor response is 40.3%. The sensitivity increases with washing in all cases. As the fabric is washed, the mechanical and chemical stress likely removes material and removes conductive pathways increasing Δ*R/R*, which is proportional to the sensor response. For use in a commercial environment the sensor response could be modelled as a logistic function, Δ*R/R* = *L*/(1 + *e*^−*kx*^) where *x* is the wash cycle number, *k* constant associated with the rate the responsivity increases and *L* is the limit at which Δ*R/R* saturates as seen in Fig. 4e. For both the RGO and MoS_2_/RGO sensor *k* ∼ 0.3 and is likely related to the rate at which poorly adhered flakes fall off the textile, while *L* ∼ 38 for the RGO sensor and *L *∼ 17 for the MoS_2_/RGO sensor and is likely related to the charge transfer between the gas analyte and the network of strongly adhered flakes to the textile. The reduced *L* of the RGO/MoS_2_-coated fabric compared to the RGO sensor, would imply that the charge transfer between gas analyte and the sensor is of a reduced magnitude compared to RGO^51^. Alternatively, using a model can be avoided by pre-washing the sensors for 50 cycles before integration in clothes so that the limit of the sensor response is always reached, and the Δ*R/R* output will be constant. Assuming an expected lifetime of a shirt of about 50 washes (one wash a week for a year), the sensor can last the entire lifetime of the shirt.

Besides being robust, it is also important for a gas sensor to recover its pre-exposure state after a given amount of time. The sensor recovery (% return to the pre-exposure state after 2 h) was investigated for both fabric sensors (Fig. 4c). Both had the highest recovery at zero wash—94.2% and 100% for RGO and RGO/MoS_2_-coated fabrics, respectively. The recovery reduced after washing cycles 1 and 5 to 89.9% and 85.3% for the RGO-coated fabric and 97.9% and 95.9% for the RGO/MoS_2_-coated fabric. In all cases, the recovery of the RGO/MoS_2_-coated fabric is higher than that of the RGO-coated fabric in humid air. The recovery patterns could also help in correcting for hysteresis in the sensor. Increasing RH steadily from 0 to 65% will increase the sensor resistance beyond the initial (R_a_) resistance value, however, this can be corrected for by calibrating the sensor response based on the characteristic experimental relationship shown in Fig. 3d.

After the afore discussed scientific investigations, the coated fabric was integrated with an DFRobot’s Bluno beetle V1.1, an Arduino-based microcontroller platform with Bluetooth^®^ Low Energy capability shown in Fig. 4f. Measurements can be taken in the ambient atmosphere irrespective of humidity and temperature variations once the sensor is calibrated. The data collection proceeds over Bluetooth^®^ on mobile phone via a mobile app developed for Android platforms based on DFRobot's Bluno Beetle app. Other components of the integrated system include a portable battery and a variable resistor. A device-level integration in a non-intrusive platform has the potential to enable democratization of air quality data by providing access to personal exposure information at the fingertips of every citizen. This device could then be integrated with other sensors for multi-parameter monitoring on a textile platform.

## Discussion

A highly sensitive RGO and MoS_2_ coated textile-based NO_2_ sensor has been reported with demonstrated sensitivity down to 20 ppb NO_2_ concentration and LOD of 2–7 ppb, one of the lowest recorded LOD for 2D material gas sensing. The sensor demonstrates its capability to withstanding up to 50 ISO-standard wash cycles and shows considerable improvement (~ 500%) in the sensor response as a result of 50 wash cycles. Also, the fabricated sensor shows selectivity towards NO_2_ when compared with CO_2_ and NH_3_. We operate our sensors in ambient temperatures (20 °C) which has competitive advantage against competing technologies such as heated metal oxides requiring much higher temperatures via embedded heaters, and which is of great importance for applications on fabrics or multibody area networks. Finally, the potential integration allowing for real-time personal exposure monitoring from a mobile application using an Arduino-compatible platform is also demonstrated.

## Methods

### Preparation of 2D material inks

In this study, we use three inks, a commercially available Graphene Oxide (GO) ink from Sigma Aldrich, a MoS_2_ ink prepared via sonication of MoS_2_ powder and a MoS_2_/GO ink prepared by mixing the GO and MoS_2_ ink in a 10:1 ratio by volume. The GO ink was bought commercially from Sigma Aldrich (part number 777676). An MoS_2_ ink was prepared by adding 10 mg ml^−1^ MoS_2_ powder (Sigma Aldrich) with 5 mg ml^−1^ sodium deoxycholate (SDC) as a stabilisation agent in deionised water. The dispersion was sonicated (Fisherbrand FB15069, Max power 800 W) for 8 h to enable the exfoliation of the bulk MoS_2_ into nanoplatelets. The dispersion was then centrifuged at 3000 rpm for 20 min to sediment the bulk MoS_2_. The supernatant (i.e. top 70%) was extracted to create the MoS_2_ ink.

### Characterisation of 2D material inks

A contact angle apparatus (First Ten Angstroms) is used to measure the surface tension of the GO and MoS_2_ ink utilising the pendant drop method. In this method, a drop is dispensed from a needle and a camera is used to image the pendant droplet resulting from the relationship between the liquid surface tension and gravity. The surface tension is calculated from the pendant drop using drop shape analysis. The inks had a surface tension of 70.02 mN/m, 48.28 mN/m and 65 mN/m for the GO, MoS_2_ and GO/MoS_2_, respectively. A parallel plate rotational rheometer (DHR rheometer TA Instruments) is used to evaluate the viscosity as a function of shear rate, the infinite-rate viscosity is found for each ink. The inks had a viscosity of 7.07 mPa s, 0.74 mPa s and 4.69 mPa s for the GO, MoS_2_ and GO/MoS_2_ inks respectively.

### ***Fabrication of GO-coated and GO/MoS***_***2***_***-coated nylon fabric***

We coated the fabric by a modified dip-coating method. Nylon fabric made from Nylon 66 and originally used as peel ply for composite manufacturing was obtained from a commercial supplier in 15 cm by 15 cm sample dimensions. The as-purchased fabric was cut to smaller (5 cm × 5 cm), fully immersible size and immersed in the GO dispersion, shaken vigorously for a few seconds, and then left to soak for 24 h. The wet, coated fabric was then dried by stapling on four corners to a plastic bag (more hydrophobic material) and hanging vertically to a cloth line in ambient air. The sample was visually inspected to confirm uniform coating. For the GO/MoS_2_-coated fabric, 15 ml GO dispersion (4 mg ml^−1^) in water was mixed with 30 ml MoS_2_ ink. The mixture was stirred with a magnetic stirrer for 20 min before immersing the fabric in the mixture and allowing it to soak and dry according to our modified dip-coating method described earlier.

### Reduction of GO-coated fabric

The dried coated fabric was thermally reduced in a France Etuves XFLO20 vacuum oven (0 to − 1000 mbar relative pressure) with adjustable temperature control (C3000 PID electronic controller) up to 200 °C with 0.1 °C precision, measured with a PT100 probe. The oven temperature was set at 170 °C and allowed to reach the set temperature. The coated fabric was placed inside the loading tray. The oven was then set to vacuum and allowed to stay for 1 h at the same temperature after reaching vacuum. The temperature was then gradually reduced to room temperature and the atmospheric pressure was restored to the oven.

### Gas sensing tests

Gas sensing tests were carried out using an in-house gas characterisation system comprising an air-tight steel chamber housed in a Panasonic (MIR-154) cooled incubator with probes for connecting to the sensor pads, a Keithley 6487 picoammeter/voltage source for biasing and measuring the current through the sensor, a vacuum flow rates, and gas cylinders. The tests carried out spanned 9 h in each case for both dry air and humid air conditions. In every case, the RGO and RGO/MoS_2_-coated fabric were placed on the sample holder. The probes were then brought into contact with it. The separation of the probes was kept constant at 2 cm all through the test cases to ensure uniformity of test situations. The voltage used was 3 V for biasing the sensor. For the dry air tests with increasing target gas concentration, the sensor was exposed to dry air until the resistance was constant. Then short time exposures of 10 min to NO_2_, followed by dry air 30 min, and then to 20 ppb NO_2_ with concentration increased by 20 ppb each time and the cycle repeated until 100 ppb. For humid air tests, the fabric was exposed to 65% RH air and allowed to reach stability before exposure to 100 ppb NO_2_ for 10 min, and then exposed to humid air for 4 h to allow for recovery.

### Raman spectroscopy

Raman measurements were taken using the Renishaw inVia Raman microscope. First, the equipment was calibrated with silicon wafer. Next, the sample was exposed to a 514.5 nm laser for 10 accumulations. We used a laser power of about 1 mW and an objective lens of 50×.

### Scanning electron microscope (SEM)

SEM Images presented in this work were taken with the High-Resolution FEI Magellan 400 with back-scatter secondary electrons detectors, two CCDs for both stage side view and sample navigation, and capability for elemental analysis via a Bruker X-ray detector.

### ISO-standard washability tests

International Standards Organization (ISO) standard ISO 105-C06 test A1S for colour fastness to textiles was used to check the resistance of the RGO-coated fabric to domestic and commercial laundering processes. The test conditions include wash and rinse temperature of 40 °C, 0% chlorine, 0 g l^−1^ sodium perborate, and 30 min per cycle wash time. No pH adjustment was required. No souring treatment in acetic acid reagent was required. We prepared the wash liquor by dissolving 600 mg of detergent (ECE Phosphate) in 150 ml of water. The fabric was removed from the detergent solution after 1, 5, 10, 20, 50, 70, 100 cycles, a piece of the fabric was then cut and rinsed twice for 1 min each in two different portions of water at 40 °C (which was prepared by filling one of the unused cylinders in the washer with water throughout the process), and dried in air. No steel balls were used in the tests as specified in the ISO standard for delicate fabrics.

### Integrated device tests

The coated fabric was integrated with DFRobot's Bluno beetle V1.1, an Arduino-based microcontroller platform with Bluetooth^®^ Low Energy module. The data was collected over Bluetooth^®^ on a mobile phone via a mobile application developed for Android platforms.

## Data Availability

The data supporting the findings of this study are available within the paper and can be accessed at 10.17863/CAM.85441.
